# Gender and disease-inclusive nomenclature consolidation of theragnostic target, prostate-specific membrane antigen (PSMA) to folate hydrolase-1 (FOLH1)

**DOI:** 10.3389/fmed.2023.1304718

**Published:** 2024-02-09

**Authors:** Marigdalia K. Ramirez-Fort, Casey K. Gilman, Jacob S. Alexander, Barbara Meier-Schiesser, Arjan Gower, Mojtaba Olyaie, Neel Vaidya, Kiarash Vahidi, Yuxin Li, Christopher S. Lange, Migdalia Fort, Corinne Deurdulian

**Affiliations:** ^1^BioFort, Guaynabo, Puerto Rico; ^2^Veterans Affairs (VA) Greater Los Angeles Healthcare System, Veterans Health Administration, United States Department of Veterans Affairs, Los Angeles, CA, United States; ^3^University of California at Los Angeles (UCLA) Health System, Los Angeles, CA, United States; ^4^San Juan Bautista School of Medicine, Caguas, Puerto Rico; ^5^University Hospital Zürich, Zürich, Switzerland; ^6^Department of Radiology, SUNY Downstate Health Sciences University, Brooklyn, NY, United States; ^7^University of Southern California, Los Angeles, CA, United States

**Keywords:** folate hydrolase-1, prostate-specific membrane antigen, theragnostic target, gender-inclusive language, disease-inclusive language

Gender and disease-exclusive language in healthcare may pose a problem in recognition or evaluation of various disease processes. Although more recognition has been placed on the use of proper pronouns in the medical literature for patient care, efforts to utilize inclusive language for biological markers, diagnostic procedures, society guidelines, and other medical terminology should be made. The continued use of gender-exclusive language in oncology and other fields may lead to the exclusion of genotypic female patients from certain therapeutic and diagnostic opportunities that prescribe masculine descriptors. One example highlighted throughout this editorial is “prostate-specific membrane antigen” or “PSMA,” which is encoded by the folate hydrolyase-1 (FOLH1) gene and is expressed in numerous solid tumors present in both male and female patients (1–9). Histopathological findings have been corroborated with molecular imaging (10).

Theragnostic agents targeting folate hydrolase-1 (FOLH1), encoded by the FOLH1 gene, have been approved by the Federal Drug Administration (FDA). FOLH1 is a transmembrane receptor and enzyme that has been linked to disease-free survival, tumor size, metastatic progression, and overall survival in a number of different tumor histologies (1). The clinical potential for targeting FOLH1 was first described in the 1980s; the analogous but membrane-bound upregulation of FOLH1 to prostate-specific antigen in the setting of prostate cancer (PCa) established the term “prostate-specific membrane antigen” at that time. In 1997, Neil H. Bander's team discovered that FOLH1 was also expressed in the neo-endothelium of melanoma, renal cell, urothelial, colon, lung, and breast carcinomas (2). In 1998, the enzyme named “PSMA” was found to be encoded by the FOLH1 gene, indicating that PSMA and FOLH1 were and are the same molecules (1, 11).

Physiologically, FOLH1 is expressed on the apical/luminal surface of the duodenum (12) (Figure 1) by proximal renal tubular cells, the prostate gland, parotid glands (Figure 2), and a subpopulation of astrocytes. It is well-known that salivary glands show avid uptake of PSMA radiopharmaceuticals with molecular imaging studies (Figure 2). Yet, several immunohistochemistry studies observed the absence of PSMA expression in salivary glands (13). Currently, it is hypothesized that PSMA radiopharmaceutical uptake in salivary gland tissue is mostly non-specific (14). Pathologically, FOLH1 is expressed on the surface membrane of PCa cells and adenoid cystic carcinoma cells, intracellularly in a subset of OCT4^+^ melanoma cells (possible cancer stem cells), and luminally in virtually every solid tumor-associated vasculature (1).

**Figure 1:**
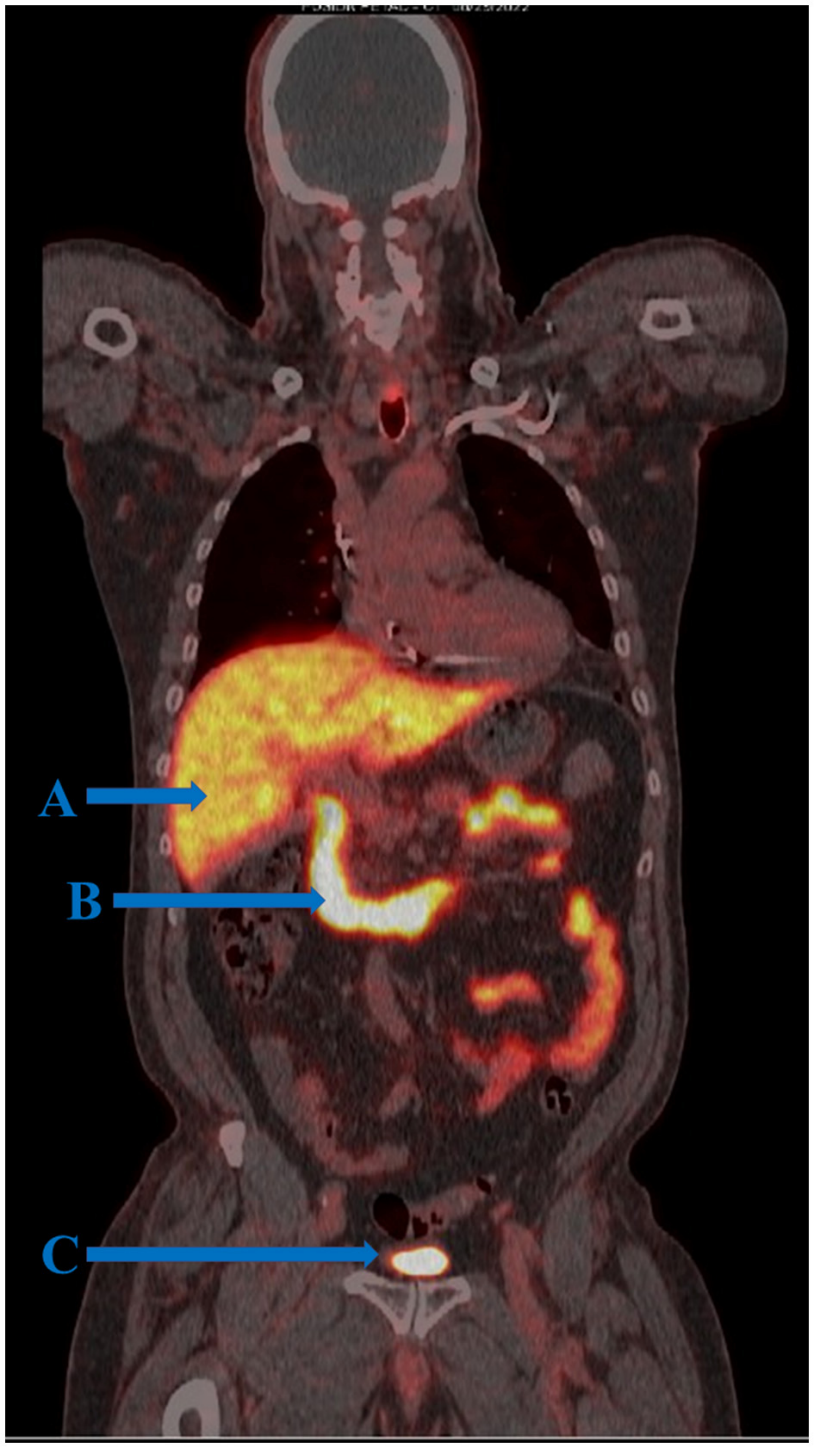
FOLH1 PET/CT of a male patient with prostate cancer. **(A)** Tracer uptake is demonstrated in the liver where tracer is metabolized and excreted into the gastrointestinal tract. **(B)** Physiologic tracer uptake is seen in the first three portions of the duodenum and small intestine where FOLH1 regulates intestinal absorption of dietary folate. Although it is known that the duodenum expresses targetable FOLH1, it is not well understood the extent of overlap between physiologic FOLH1-mediated tracer uptake and background positron capture from tracer in transitory bile. **(C)** Tracer activity in the bladder.

**Figure 2:**
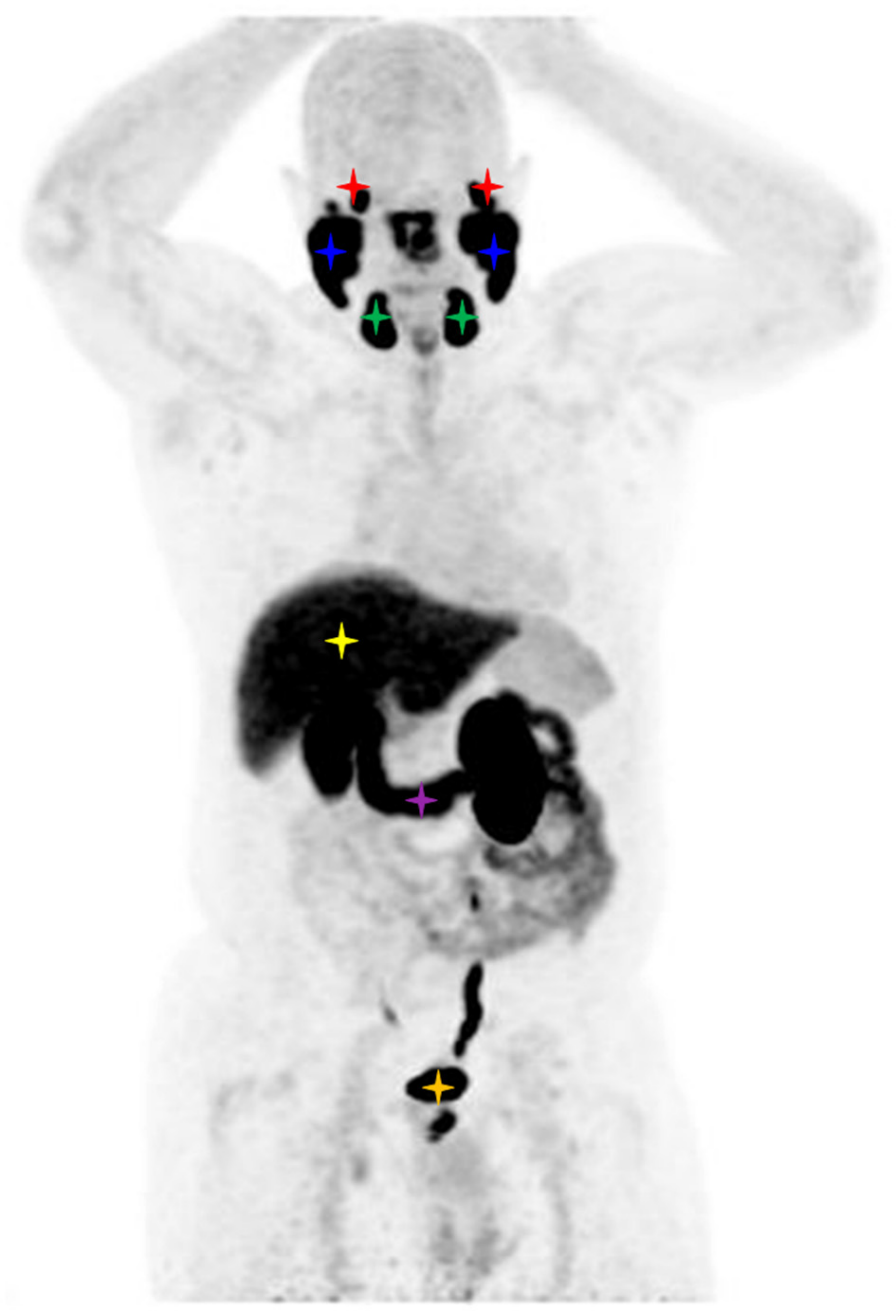
Maximum intensity projection image of a FOLH1 PET/CT of a male patient with prostate cancer. Physiological tracer uptake is seen in the lacrimal glands (red stars), parotid glands (blue stars), submandibular glands (green stars), liver (yellow star), duodenum (purple star), and bladder (orange star).

Nguyen et al. (15) demonstrated that the substrate of solid tumors *in vitro* induces neoendothelial FOLH1 expression in preclinical solid tumor models containing human umbilical vein endothelial cells. This study demonstrated that FOLH1 is specific to the formation of tumor-associated vessels rather than angiogenic vessels of other etiology (15). Further, our group recently validated FOLH1 as a novel theragnostic target in Merkel cell carcinoma; specifically, prevalent FOLH1 expression within Merkel cell carcinoma-associated neovessels, in 60–77% of patients in an 81-person cohort (3).

Different aliases such as glutamate carboxypeptidase II, N-acetyl-L-aspartyl-L-glutamate peptidase, FOLH1, and PSMA are currently used interchangeably in the medical literature. The foremost names attempt to title the enzyme primarily based on the location and enzymatic function in which it was discovered (i.e., kidney, nervous system glia, duodenum, and the small bowel). For example, glutamate carboxypeptidase II and N-acetyl-L-aspartyl-L-glutamate peptidase are typically described as being found in astrocytes where they cleave terminal glutamate residues to form the neurotransmitter glutamate (1). On the other hand, the use of the PSMA is solely based on its most common contemporary medical use (i.e., FDA-approved PSMA PET/CT and/or 177Lu PSMA molecular brachytherapy).

Decades of oncological research have focused on characterizing FOLH1 expression in PCa; therefore, it is understandable that the most common terminology used to cite this theragnostic target in oncology is “PSMA,” suggesting exclusive enzyme expression to the prostate. The continued use of PSMA, particularly in society guidelines, is problematic and should be considered a misnomer as it falsely implies exclusive expression to the prostate (16) [i.e., the European Association of Nuclear Medicine (EANM) standardized reporting “guidelines v1.0 for PSMA-PET” state “PSMA is a glycoprotein, a membrane-bound metallo-peptidase, encoded by FOLH1 gene on chromosome 11”]. Based on the broader potential of oncological use and clinical significance in a variety of non-prostatic malignancies, accurate nomenclature should be established and standardized with urgency to optimize ongoing translational cancer research and to avoid confusion amongst physicians and patients.

Little effort has been made to standardize the nomenclature to a more representative description that is readily applicable to cancer in other organ systems. For example, groups have reported on FOLH1 expression in the stroma of Merkel cell carcinoma (3) and transitional cell carcinomas of the bladder (4), while other groups reported the same enzyme as “PSMA” expression in melanoma and osteosarcomas (5, 6), demonstrating the lack of consolidated nomenclature in various medical fields managing distinct organ systems. Furthermore, the widely cited PSMA misnomer generates a gender bias that could translate into reduced diagnostic and therapeutic opportunities for genotypic female patients with FOLH1-expressing solid tumors. Misleading nomenclature and related gender bias could adversely impact future FDA approvals for theragnostic indications for all solid tumors that express FOLH1 in the neovasculature, including for gynecological malignancies in patient genotypes that do not have prostates (7).

“FOLH1,” however, is a strong candidate for officiating nomenclature as its name does not imply exclusivity to certain tissues or biological sexes but rather describes its enzymatic function. There is a strong international movement for the use of gender-inclusive language in many aspects of the modern world beyond medicine and science, such as in government and labor (e.g., United Nations Global Sustainable Development Goals). To this end, the U.S. General Services Administration 18F Content Guide strongly suggests that formal writings that include words and phrases that indicate gender bias should be avoided. Similar suggestions should be applied in both science and medicine. Furthermore, the International Union of Biochemistry and Molecular Biology has established that the scientific nomenclature of enzymes should be based on the chemical reaction that they catalyze, as this is a unique feature of each enzyme (17). Inherent to its name, “folate hydrolase-1” utilizes water to break down (i.e., hydrolysis) glutamate residues from dietary folate. Therefore, consolidating the nomenclature into the gene, FOLH1, that encodes the enzyme, is a practical method of standardization.

Regardless of the challenges that a change in nomenclature may pose, if we aspire to create scientific unity, we should collectively consider establishing a common language as delineated by the guidelines set forth by the International Union of Biochemistry and Molecular Biology and the United Nations Global Sustainable Development Goals. The authors recommend establishing gender and disease-inclusive nomenclature for this important biomarker and theragnostic target, encoded by the FOLH1 gene, as “FOLH1.”

## Author contributions

MR-F: Conceptualization, Data curation, Formal analysis, Visualization, Writing – original draft, Writing – review & editing. CG: Writing – original draft, Writing – review & editing. JA: Writing – original draft, Writing – review & editing. BM-S: Writing – review & editing, Conceptualization, Supervision, Writing – original draft. AG: Writing – review & editing. MO: Writing – review & editing. NV: Writing – review & editing. KV: Writing – review & editing. YL: Supervision, Writing – review & editing. CL: Supervision, Writing – review & editing, Conceptualization, Formal analysis. MF: Resources, Supervision, Writing – original draft, Writing – review & editing. CD: Supervision, Validation, Writing – review & editing.
